# *Tephrosia toxicaria* (Sw.) Pers. extracts: Screening by examining aedicidal action under laboratory and field conditions along with its antioxidant, antileishmanial, and antimicrobial activities

**DOI:** 10.1371/journal.pone.0275835

**Published:** 2023-01-11

**Authors:** Giulian César da Silva Sá, Leidiane Barboza da Silva, Pedro Vitor Vale Bezerra, Melissa Alves Farias da Silva, Cássio Lázaro Silva Inacio, Weslley de Souza Paiva, Virgínia Penéllope Macedo e Silva, Laísa Vilar Cordeiro, Johny Wysllas de Freitas Oliveira, Marcelo Sousa Silva, Edeltrudes de Oliveira Lima, Francisco José Carvalho Moreira, Hugo Alexandre de Oliveira Rocha, Patricia Batista Barra, Maria de Fátima Freire de Melo Ximenes, Adriana Ferreira Uchôa

**Affiliations:** 1 Department of Cellular Biology and Genetics, Universidade Federal do Rio Grande do Norte, Natal, Rio Grande do Norte, Brazil; 2 Instituto de Medicina Tropical do Rio Grande do Norte, Natal, Rio Grande do Norte, Brazil; 3 Department of Microbiology and Parasitology, Laboratory of Entomology Research, Universidade Federal do Rio Grande do Norte, Natal, Rio Grande do Norte, Brazil; 4 Department of Biochemistry, Laboratory of Biotechnology of Natural Polymer, Universidade Federal do Rio Grande do Norte, Natal, Rio Grande do Norte, Brazil; 5 Department of Pharmaceutical Sciences, Laboratory of Mycology, Universidade Federal da Paraiba, João Pessoa, Paraiba, Brazil; 6 Department of Clinical and Toxicological Analysis, Laboratory of Immunoparasitology, Universidade Federal do Rio Grande do Norte, Natal, RN, Brazil; 7 Laboratory of Seeds Plant Health, Instituto Federal de Educação, Ciência e Tecnologia do Ceará, Sobral, Ceará, Brazil; 8 Department of Biomedical Sciences, Universidade do Estado do Rio Grande do Norte, Mossoró, Rio Grande do Norte, Brazil; Al-Azhar University, EGYPT

## Abstract

An increase in the incidence of arboviral, microbial and parasitic infections, and to disorders related to oxidative stress has encouraged the development of adjuvant therapies based on natural formulations, such as those involving plant extracts. Thus, to expand the repertoire of the available therapeutic options, this study aimed to describe the versatility of *Tephrosia toxicaria* (Sw.) (Pers., 1807) extracts for the control of arbovirus vectors, as well as their antioxidant, antileishmanial, and antimicrobial potential. Among the aqueous and hydroethanolic extracts obtained, the hydroethanolic extract from roots (RHA) was identified as the most active larvicide extract demonstrating, respectively, the lowest lethal concentration (mg/mL) for 50%, 90% and 99% of *Aedes aegypti* (L., 1762) and *Aedes albopictus* (S., 1894) larvae, observed at 24 h (0.33, 0.84 and 1.80; 0.32, 0.70 and 1.32) and 48 h (0.17, 0.51 and 1.22; 0.26, 0.47 and 0.78) post-exposure. Field assays revealed that RHA (0.84 mg/mL) is a potential oviposition deterrent, reducing egg-laying by approximately 90%. RHA (0.1 mg/mL) also exhibited antioxidant activity for the following tests: total antioxidant capacity (286.86 mg AAE/g), iron (87.16%) and copper (25.64%) chelation, and superoxide scavenging (10%). In the cell culture assays, RHA (0.1 mg/mL) promoted regeneration of metabolic activity (92% cell viability) in cells exposed to oxidative stress. Furthermore, RHA displayed weak antileishmanial activity (IC_50_ = 3.53 mg/mL) against *Leishmania amazonensis* and not exhibit antimicrobial activity. The extraction favored the concentration of carbohydrates in RHA, in addition to lectins and protease inhibitors, with molecular masses estimated between 10 and 24 kDa. Cytotoxicity and phytotoxicity analyses of RHA suggested its biosecurity. Thus, RHA is a multivalent extract with insecticide and antioxidant properties at low and safe concentrations. However, others studies on its indirect toxic effects are ongoing to ensure the complete safety of RHA.

## Synopsis

Despite efforts to control cases of arboviral diseases (arthropod-borne viral), microbial, and parasitic infections, or to delay the deleterious effects of oxidative stress, highly efficacious treatments are lacking, and the emergence of resistance to insecticides and antibiotics is increasing. Natural adjuvant formulations, such as plant extracts, have been explored to support integrated disease and vector management systems. Plant extracts are promising sources of biologically active molecules that are recognized for their complex modes of action, enabling the exploitation of such extracts for various purposes. This study aimed to elucidate the biological versatility of the extracts of *Tephrosia toxicaria*, a legume widely distributed worldwide and occurring in northeast Brazil, popularly known as “timbó de caiena”. Among the eight extracts obtained from seeds, roots, stems and leaves of *T*. *toxicaria*, hydroethanolic extract from roots (RHA) was identified to be safe (without toxicity against cell lines and non-target organisms) and exhibited bioactivity, i.e., insecticide and antioxidant properties at low concentrations. These results may expand the biological relevance of vegetation in northeastern Brazilian biomes. Study authors suggest that *T*. *toxicaria* and its extracts can be exploited to assist vector and disease management programs, improving the health and quality of life of the global population.

## Introduction

In complementary and alternative medicine, plants remain a potential source for the development of natural formulations, such as plant extracts, to prevent and treat numerous diseases worldwide [1, 2]. Plant extracts are ecologically sustainable, less harmful to non-target organisms compared to chemical insecticides, and exhibit complex compositions [3, 4]. Brazilian plant biodiversity includes more than 40,000 plant species distributed in six biomes (Amazon Forest, Caatinga, Cerrado, Atlantic Forest, Pantanal and Pampa) for possible sustainable exploitation [5]. Amidst such plant diversity, the *Tephrosia* Pers. (Fabaceae, subfamily Paplilionaceae) genus is a perennial genus derived from the African continent, with approximately 350 species widely distributed in tropical, subtropical, and arid regions of the world [6]. In Brazil, 12 *Tephrosia* species have been recorded, including *Tephrosia toxicaria* (Sw.) Pers., a North American legume (shrubby habit) well adapted to Northeast Brazil [7, 8]. *Tephrosia* extracts are already being explored to control numerous species of arbovirus vectors and field pests [9, 10], in addition to the possibility of overcoming the concerns of numerous international health agencies, such as damage associated with oxidative stress, and bacterial, fungal and parasitic infections [11–13].

Arboviruses (viruses transmitted by arthropod vectors) account for more than 17% of all viral infections, with *Aedes aegypti* (Linnaeus, 1762), *Aedes albopictus* (Skuse, 1894) and Phlebotomus sandflies as remarkable models of viral competence owing to their seasonal transmission and sensitivity to climatic factors, requiring measures to constantly control their global spread [14, 15]. Although arboviruses account for more than 390 million infection cases per year and more than 20,000 lethal cases, mainly in tropical countries [16], immunization protocols against all known arboviruses are lacking, for example, yellow fever virus [17]. Current health policies in vector control commonly include mechanical control and the use of chemical insecticides [18]. However, mechanical control has numerous limitations, and the unrestricted use of chemical insecticides can lead to resistance among insects [18] and induce toxicity in non-target organisms, including humans [19]. Hence, new and safe strategies, such as insecticidal plant extracts, must be implemented in vector management systems [20].

Oxidative stress is an important factor in the etiology and progression of numerous metabolic [21] and neurodegenerative syndromes [22], in addition to aging and the onset of age-related diseases [23]. Numerous studies have also confirmed the impact of the severity of bacterial, fungal and leishmanial infections in various industrial sectors and international health systems [24–26]. Damage control associated with oxidative stress is primarily attributed to endogenous antioxidant systems. Antibiotics and pentavalent antimonials are the first-line therapies for the treatment of microbial infections and leishmaniasis, respectively. However, due to severe side effects in patients and the emergence of resistance mechanisms in pathogens due to incorrect and/or excessive use of antimicrobials and due to the inadequacy of fully effective antioxidant therapies, safe and effective adjuvants must be developed and used [26–28]. Adjuvant therapy with natural formulations has been employed for many decades. In fact, since 1981, approximately 40% of the drugs approved by the U.S. Food and Drug Administration (FDA) have been derived from natural sources, including plants [2].

To expand the access of plant formulations as adjuvants in the integrated management of vectors and diseases, this study aimed to examine the versatility of *T*. *toxicaria* extracts in the control of *A*. *aegypti* and *A*. *albopictus* under laboratory and field conditions, determine their antioxidant, antimicrobial, and antileishmanial potential, and evaluate their toxic effects against non-target organisms and cell lines.

## Materials and methods

The project was approved by the UFRN Research Ethics Committee and registered on Plataforma Brasil (CAAE: 45646421.0.0000.5537). The participants (healthy adults) signed a consent form.

### *Tephrosia toxicaria* extracts

*Tephrosia toxicaria* was collected in São José district, Ibiapina-CE, Brazil (3°55’31.0" S 40°53’43.0" W), under SisGen (Brazilian National Management System Genetic Heritage and Associated Traditional Knowledge) regulations (SisGen n. A3104E7). A specimen (voucher UFRN00022960) was deposited at the Herbarium of Universidade Federal do Rio Grande do Norte (UFRN), Natal-RN, Brazil. Seeds, roots, stems and leaves of *T*. *toxicaria* were dried (±37°C) in a circulating air environment and grinding (30 mesh) in a Willye knife mill model STAR FT-50 (Fortinox, Brazil). For obtaining hydroethanolic extracts, 100 g of each flour was submitted to extraction with 500 mL of 80% ethanol P.A. (Synth, Brazil), remaining at rest for 24 h, with subsequent centrifugation (10,000 x g, 4°C, 30 min) (Centrifuge model 5810R, Eppendorf, Germany). Aqueous extracts were obtained under the same conditions, replacing ethanol with distilled water. The extracts were lyophilized (FreeZone4.5 freeze dryer, Labcon, USA) and named as follows: SAQ (aqueous extract from seeds), SHA (hydroethanolic extract from seeds), RAQ (aqueous extract from roots), RHA (hydroethanolic extract from roots), StAQ (aqueous extract from stems), StHA (hydroethanolic extract from stems), LAQ (aqueous extract from leaves) and LHA (hydroethanolic extract from leaves).

### Insecticide activity

For laboratory assays, *A*. *aegypti* and *A*. *albopictus* larvae were from the colony established at the Laboratory of Entomology, Department of Microbiology and Parasitology, UFRN, Natal-RN, Brazil. Adult insects were collected in different municipalities in the state of Rio Grande do Norte, Brazil, and were kept in breeding cages at a constant temperature of 28°C and natural photoperiod, and fed a sugary solution (10%). Every 48h, a hamster (*Mesocricetus auratus*) was offered to females for a blood meal. Oviposition occurred in ovitraps placed in the cages. The hatched larvae were used in larvicidal trials, according to WHO [29] guidelines. Randomly, 20 larvae were transferred to glass beakers containing 20 mL of crude extracts (1.25–25% v/v), distilled water (control) or ethanol (vehicle control). The ethanol concentration was adjusted in the dose-response experiments, following the same dilution parameter for the crude extracts. Larvae survival was evaluated after 24h and 48h of incubation, and larvae that did not respond to mechanical stimuli were considered dead. Morphological alterations in larvae were visually examined through photos taken under a magnifying glass (Zeiss Stemi 508 stereomicroscope) to make a comparison between the different treatments. Lethal concentrations (LC_50_, LC_90_ and LC_99_) were expressed in mg/mL, based on the dry weight of extracts. The extract with lower LC values was selected to perform the other tests. Ethanol was completely removed from the extract and subsequent assays were performed with the lyophilized extract (doses were expressed in mg/mL).

The insecticide effects under field conditions were evaluated according to Fay & Eliason [30]. Black plastic containers (1.5L capacity; Ovitrap) were placed randomly under trees in three coverage areas located in the municipality of Mossoró-RN, Brazil (5°12’17.8"S 37°18’55.8"W). The municipality is approximately 285 kilometers (177 miles) from Natal-RN, the State capital, and has a population of 303,792 inhabitants [31]. Its climate is dry and the average temperature and humidity during the months of execution of the field tests (January to March 2020), respectively, were 27.8°C (27.3°C– 28.3°C) and 77.1% (73.9%– 80.3%) [32]. A pressed wood pallet (2.5 cm x 15 cm; Eucatex^®^) was attached to each ovitrap by means of a staple, leaving the roughened part of the wood pallet exposed to the inside of the ovitrap. For six weeks, ovitraps containing only water (control; 300 mL) were monitored and from the seventh week onwards, ovitraps containing extract (300 mL) were added to the monitoring. Positivity, egg density and number of eggs per ovitrap were recorded at weekly intervals for the calculation of the egg density index (EDI) and oviposition positivity index (OPI), according to Moura et al. [33]. EDI is the average number of eggs per ovitrap, obtained by dividing the number of eggs found and the number of ovitraps evaluated. OPI is obtained by dividing the number of positive ovitraps and the number of examined ovitraps.

### Antioxidant activity

Total antioxidant capacity (TAC) of *T*. *toxicaria* extracts (0.1 mg/mL) was evaluated by phosphate-molybdate complex reduction method [34]. The TAC values were expressed as ascorbic acid equivalents (AAE/g). The reducing power of *T*. *toxicaria* extracts (0.1, 0.5 and 1.0 mg/mL) was examined by potassium ferricyanide reduction assays [35] and results were expressed as the percentage of activity for 0.1 mg/mL (highest activity) of ascorbic acid. The copper- and iron-chelating activities of *T*. *toxicaria* extracts (0.1, 0.5 and 1.0 mg/mL) were determined according to Anton [36] and Dinis et al. [37] methods, respectively. The results of the chelating activities were expressed as the percentage of chelating effect, using the following Equation: Chelating Effect (%) = [(Ab–Ae) / Ab] x 100, where Ab (Absorbance of blank) and Ae (Absorbance of the extract). The hydroxyl radical- and superoxide radical-scavenging activities of *T*. *toxicaria* extracts (0.1, 0.5 and 1.0 mg/mL) were determined according to Dasgupta & De [38] and Beauchamp & Fridovich [39] methods, respectively. The results were expressed as the percentage of hydroxyl and superoxide radical-scavenging activities, according to the following Equation: Scavenging Effect (%) = [(Ac–Ae) / (Ac–Ab)] x 100, where Ac (absorbance of control), Ae (absorbance of extract) and Ab (absorbance of blank).

Antioxidant activity of *T*. *toxicaria* extracts in cell culture was evaluated by the 3-(4,5-dimethylthiazol-2-yl)-2,5-diphenyltetrazolium bromide (MTT) (Merck, Germany) reduction method [40]. After determining the injury condition (H_2_O_2_-induced oxidative stress), according to Ouyang et al. [41], murine fibroblast cell lines (3T3, ATCC^®^ CRL-1658™) were exposed to different concentrations of H_2_O_2_ (0.5–5.0 mM) (CRQ, Brazil). As a result, at a H_2_O_2_ concentration of 4 mM the cells suffered enough damage to decrease MTT expression by up to 45% (positive control). Concentrations greater than 4 mM caused damage well over 45%, while the negative control (without H_2_O_2_) showed no damage. The 3T3 cells were initially exposed to H_2_O_2_ (4 mM) for 1 h and later treated with the *T*. *toxicaria* extracts (0.001, 0.01 and 1.0 mg/mL) for 24 h. The absorbance (570 nm; BioTek Instruments Inc., USA) of the control without H_2_O_2_ was considered to be a 100% reduction in MTT assay and the values of the treated cells were calculated as a percentage of the negative control, without H_2_O_2_. Results were expressed as the percentage of MTT reduction, according to the following Equation: MTT Reduction (%) = [(Ae / Ac) x 100], where Ae (absorbance of cells subjected to treatment with extracts) and Ac (absorbance of cells from the negative control).

### Antileishmanial activity

*Leishmania amazonensis* (MHOM/BR/73/M2269) were obtained from the parasitological collection of the Immunoparasitology Laboratory, Department of Clinical and Toxicological Analysis, UFRN, Natal-RN, Brazil. Promastigote forms of *L*. *amazonensis* were cultivated in RPMI 1640 medium (Sigma-Aldrich, USA), at 27 ± 2°C for 4 days until the log phase. A volume of 200 μL of a parasitic inoculum (10^7^ parasites/mL) was added to extracts (0.1, 0.5 and 1.0 mg/mL) in a 96 well-plate, and incubated for 24 h at 27 ± 2°C. Parasitic viability was measured by the resazurin reduction assay. Briefly, 20 μL of 1 mM of resazurin (Sigma-Aldrich, USA) was added to the plates after each incubation time and then incubated again for 24 h. Absorbance was determined at 570 and 600 nm in a microplate reader (Epoch-Biotek, USA) [42]. The percentage of inhibition was calculated using the following Equation: Parasitic Inhibition (%) = [100 − ((A570t − (A600t × R0)) / (A570c − (A600c × R0))) × 100], where A570t: Absorbance of the treatment at 570 nm; A600t: Absorbance of the treatment at 600 nm; A570c: Absorbance of the control at 570 nm; A600c: Absorbance of the control at 600 nm. R0: Correction factor of the influence of the media on the resazurin reduction, the product of absorbance of the media at 570 nm to the absorbance of the media at 600 nm.

### Antimicrobial activity

Bacteria (*Bacillus subtilis* ATCC-6633, *Escherichia coli* ATCC-25922, *Klebsiella pneumoniae* ATCC-700603, *Proteus mirabilis* ATCC-25933, *Pseudomonas aeruginosa* ATCC-9027, *Salmonella enterica* ATCC-13076, *Staphylococcus aureus* ATCC-25923 and *Staphylococcus epidermidis* ATCC-12228), yeast (*Candida albicans* ATCC-90028, *Candida krusei* ATCC-6258, *Candida parapsilosis* ATCC-22019 and *Candida tropicalis* ATCC-13803) and filamentous fungi (*Aspergillus flavus* ATCC-9643, *Aspergillus fumigatus* ATCC-40640, *Penicillium citrinum* LM-918 and *Rhizopus oryzae* LM-4557) were obtained from the microbiological collection housed in Laboratory of Mycology, Department of Pharmaceutical Sciences of the Universidade Federal da Paraiba (UFPB), João Pessoa-PB, Brazil.

The fungal and bacterial strains were maintained at 4°C in Sabouraud Dextrose Agar (Difco Laboratories Ltd., USA) and Brain Heart Infusion (Difco Laboratories Ltd., USA), and incubated at 35 ± 2°C for 24–48 h, respectively. The microorganism suspension was prepared according to the 0.5 McFarland scale tube and was adjusted by the use of a spectrophotometer (530 nm) to 90% T [43–46]. The minimum inhibitory concentration (MIC) was determined by the micro dilution technique (1024–32 μg/mL) [46, 47]. The bacterial growth was accompanied by the colorimetric change of the 0.01% resazurin dye (INLAB, Brazil). The MIC was defined as the lowest concentration of *T*. *toxicaria* extract capable of visually inhibiting microbial growth with no dye color change. Chloramphenicol (100 μg/mL; Sigma-Aldrich, USA) was the negative control for bacterial assays, and nystatin (100 μg/mL; Sigma-Aldrich, USA) and fluconazole (50 μg/mL; Sigma-Aldrich, USA) were the negative controls for yeast and filamentous fungi, respectively.

### Cytotoxicity assays

The cytotoxicity of *T*. *toxicaria* extracts (0.001, 0.01, 0.1 and 1.0 mg/mL) was evaluated using Mosmann’s [40] method. Hepatocellular carcinoma cells (HepG2, ATCC^®^ HB8065™) and murine fibroblast cells (3T3, ATCC^®^ CRL-1658™) were donated by Dr. Viviane Souza do Amaral and Dr. Silvia Regina Batistuzzo de Medeiros, respectively, from Department of Cellular Biology and Genetics, UFRN, Natal-RN, Brazil. HepG2 and 3T3 cells (5 x 10^4^ cells) were cultivated (Incubator HEPA Filter Model 3110 Themoforma Serie II Water CO_2_, USA) in Dulbecco’s modified Eagle (DMEM; Cultilab, Brazil) and supplemented with 10% fetal bovine serum (FBS; Cultilab, Brazil), 2% L-glutamine (Sigma-Aldrich, USA) and 1% streptomycin/penicillin (Sigma-Aldrich, USA), at 37°C and 5% CO_2_. The control included cells cultivated in DMEM and 10% FBS, and was able to promote 100% reduction of MTT (1.0 mg/mL), considered as 100% of cell proliferation. Results were expressed as the percentage of MTT reduction, using the following equation: MTT Reduction (%) = (Ae–Ac) x 100, where Ae: absorbance of cells subjected to treatment with extracts, and Ac: absorbance of control.

### Phytotoxicity assays

Phytotoxicity assays on *Lactuca sativa*, cultivar “Babá de Verão” (Feltrin^®^, 98.3% purity), were performed according to USEPA [48], standard OPPTS 850.4200. *Lactuca sativa* seeds (25 seeds) were sown in Petri dishes (100 x 15 mm^2^) containing filter paper (Ø 90 mm, Whatman No. 3 filter) soaked with 5 mL of *T*. *toxicaria* extracts (0.001, 0.01, 0.1 and 0.3 mg/mL) and incubated in a BOD-type germination chamber (25 ± 3°C; relative humidity: 70%; photoperiod: 16 hours light/8 hours dark). Sterile distilled water was the positive control. Seventy-two hours after sowing, the first germination count was performed, considering the minimum emergence of 1 cm of the radicle as germination. Seven days after sowing, biomass was determined on an analytical balance (precision of the scale = 0.0001g), radicle and seedling length (cm) were measured and the percentage (%) of relative seed germination, relative radicle growth and germination index were determined according to Tam & Tiquia [49]. Normalized residual percentage of germinated seeds index (NRPGSI) and normalized residual elongation root index (NRERI) were determined according to González et al. [50]. NRPGSI and NRERI indicate the following toxicity level: 0 to -0.25: low; -0.25 to -0.5: moderate; -0.5 to -0.75: high; -0.75 to -1.0: very high; > 0: Hormese (a stimulating effect).

### Initial characterization of extracts

Total soluble protein content was measured according to Bradford [51], using bovine serum albumin (Sigma-Aldrich, Brazil) as standard, and Coomassie Brilliant Blue G-250 (Bio-Rad Laboratories Inc., USA) as chromogenic reagent. Total sugars content was quantified with the phenol-sulfuric acid method [52], using D-galactose (Sigma-Aldrich, Brazil) as the standard. Total phenolic compounds were quantified by the colorimetric method of Folin-Ciocalteu [53], using gallic acid (Casa da Química Ind. E Com., Brazil) as the standard.

The estimation of the relative molecular mass of the proteins was conducted by electrophoresis (SDS-PAGE) in the presence of 1% SDS (Sigma-Aldrich, USA), according to Laemmli [54]. The application gel was prepared in the concentration of 3.5% and the separation gel, 12%. The mass estimation was obtained by comparison to the relative electrophoretic mobility of the molecular weight standard (225–12 kDa; Amersham™).

Protease inhibitors were identified according to Xavier-Filho et al. [55], using 1% azocasein (Sigma-Aldrich, Brazil) as substrate for the following enzymes (Sigma-Aldrich, Brazil): trypsin (0.3 mg/mL), chymotrypsin (0.2 mg/mL), papain (0.2 mg/mL) and bromelain (0.3 mg/mL). The inhibitor unit (IU) was defined as the amount of inhibitor that can decrease by 0.01 the absorbance value in the protease inhibitor assay, and specific activity was considered as the relationship between IU and amount of protein used in the assay. The lectin presence was macroscopically detected by hemagglutination assays in 4% human red blood cells (blood types A, B and O) [56]. The human red blood cells were collected according to the recommendations of the Ethics and Human Research Committee of UFRN, Brazil (authorization n. 4.769.699). The results were expressed in hemagglutination units (HU), which was defined as the inverse of the highest extract dilution that showed clear agglutination.

### Statistical analysis

Data were expressed as mean ± standard deviation of three repetitions (n = 3). Lethal (LC) and cytotoxic concentrations (CC) were calculated from dose-mortality correlation in Probit, with a 95% confidence interval, using Microsoft Excel 2010 software. Analysis of variance (ANOVA) was performed on GraphPad Prism^®^ version 6.01 (GraphPad Software, USA), with Tukey’s post-test. A value of P < 0.05 was considered statistically significant.

## Results

The bioactivity of *T*. *toxicaria* extracts against *A*. *aegypti* larvae varied among the eight extracts obtained from each organ of the plant, highlighting the marked larvicidal activity of hydroethanolic extracts compared to that of aqueous extracts (Table 1). Twenty-four hours post-exposure, the RHA and LHA extracts were found to be toxic to 100% of the *A*. *aegypti* larvae when administered at a concentration of 25% (v/v). Additionally, during the same exposure period, RHA at concentrations of 12.5% (v/v) and 6.25% (v/v) was toxic to 95% and 75% of the larvae, respectively. Forty-eight hours post-exposure, RAQ, LAQ, and SHA extracts also promoted larval mortality by 100% at a concentration of 25% (v/v). StAQ did not exhibit larvicidal activity against *A*. *aegypti* larvae at any concentration or exposure time. To enable a real comparison of the active ingredients, LC values were determined and expressed in mg/mL (Table 2). Based on the overlapping 95% confidence intervals (n = 2460 larvae) for LC values, RHA was identified as the most active larvicidal extract, presenting the lowest LC_50_, LC_90_, and LC_99_ (mg/mL) values obtained at 24 h (0.33, 0.84 and 1.80 mg/mL, Slope/SE = 3.179/0.075, χ ^2^ = 0.776 and R^2^ = 0.995) and 48 h (0.17, 0.51 and 1.22 mg/mL, Slope/SE = 2.765/0.091, χ ^2^ = 0.425 and R^2^ = 0.930) post-exposure.

**Table 1:** Mortality percentage of *Aedes aegypti* larvae (L4) exposed to *Tephrosia toxicaria* extracts.

Exposure time	Extract concentration (v/v)	Larvae mortality (%) after exposure to *Tephrosia toxicaria* extracts							
		SAQ	SHA	RAQ	RHA	StAQ	StHA	LAQ	LHA
24 h	Water (control)	0	0	0	0	0	0	0	0
	80% ethanol (v/v) (vehicle control)	na	0	na	0	na	0	na	0
	1.56%	5	0	0	10	0	0	0	0
	3.12%	5	0	5	45	0	0	0	0
	6.25%	15	25	20	75	0	0	0	0
	12.5%	20	65	70	95	0	0	30	40
	25.0%	60	95	85	100	0	15	50	100
48 h	Water (control)	0	0	0	0	0	0	0	0
	80% ethanol (v/v) (vehicle control)	na	0	na	0	na	0	na	0
	1.56%	10	0	0	35	0	0	0	0
	3.12%	15	5	5	80	0	0	0	0
	6.25%	25	30	45	90	0	0	70	0
	12.5%	40	75	80	100	0	0	85	80
	25.0%	80	100	100	100	0	35	100	100

na: Not applied to aqueous extracts.

**Table 2:** LC_50_, LC_90_ and LC_99_ values for *Aedes aegypti* larvae (L4) exposed to *Tephrosia toxicaria* extracts.

Extract	LC_50_ (95% CI)	LC_90_ (95% CI)	LC_99_ (95% CI)	Slope (SE)	χ ^2^	R^2^
**24 h post-exposure**						
SAQ	16.66 (8.74–31.76)	122.49 (64.24–233.56)	623.04 (326.76–1187.97)	1.53 (0.143)	0.090	0.871
SHA	1.68 (1.24–2.26)	3.61 (2.67–4.87)	6.74 (5.00–9.09)	3.85 (0.066)	0.075	0.998
RAQ	3.15 (2.24–4.43)	8.15 (5.80–11.46)	17.69 (12.58–24.87)	3.12 (0.320)	0.406	0.984
RHA	0.33 (0.24–0.47)	0.84 (0.60–1.18)	1.80 (1.29–2.53)	3.18 (0.075)	0.776	0.995
StAQ	nd	nd	nd	nd	nd	nd
StHA	4.64 (3.29–6.56)	12.06 (8.54–17.03)	26.26 (18.59–37.09)	3.09 (0.077)	0.095	0.994
LAQ	12.42 (6.95–22.20)	67.59* (37.82–120.80)	268.93* (150.47–480.65)	1.74 (0.129)	nd	1.000
LHA	12.31 (8.88–17.05)	24.98 (18.02–34.61)	44.48 (32.10–61.64)	4.16 (0.072)	nd	1.000
**48 h post-exposure**						
SAQ	7.37 (4.30–12.64)	45.14 (26.33–77.39)	197.77 (115.35–339.07)	1.67 (0.119)	0.270	0.907
SHA	1.48 (1.10–1.99)	3.18 (2.36–4.29)	5.95 (4.41–8.02)	3.85 (0.066)	0.603	1.000
RAQ	2.22 (1.67–2.96)	4.56 (3.42–6.07)	8.18 (6.14–10.91)	4.13 (0.064)	0.261	0.984
RHA	0.17 (0.11–0.26)	0.51 (0.34–0.76)	1.22 (0.81–1.84)	2.76 (0.091)	0.425	0.930
StAQ	nd	nd	nd	nd	nd	nd
StHA	2.35 (2.07–2.67)	3.05 (2.69–3.45)	3.76 (3.32–4.26)	11.41 (0.028)	nd	1.000
LAQ	1.53 (0.90–2.60)	8.66* (5.08–14.76)	35.61* (20.88–60.71)	1.70 (0.118)	nd	1.000
LHA	4.31 (3.15–5.91)	13.45 (9.83–18.42)	34.01 (24.84–46.56)	2.59 (0.070)	nd	1.000

Values generated in Probit, with a 95% confidence interval (CI). LC_50_, LC_90_ and LC_99_ values (lethal concentration for 50, 90 and 99% of larvae, respectively) are expressed in mg/mL of extract (lower–upper). (nd) Not Determined. P = 0.1813 and 0.1616 in all cases, for 24- and 48 h post-exposure, respectively, except in *, where P = 0.05. Values within a row and time exposure followed by the * do not differ based on overlapping 95% CIs.

**Fig 1:**
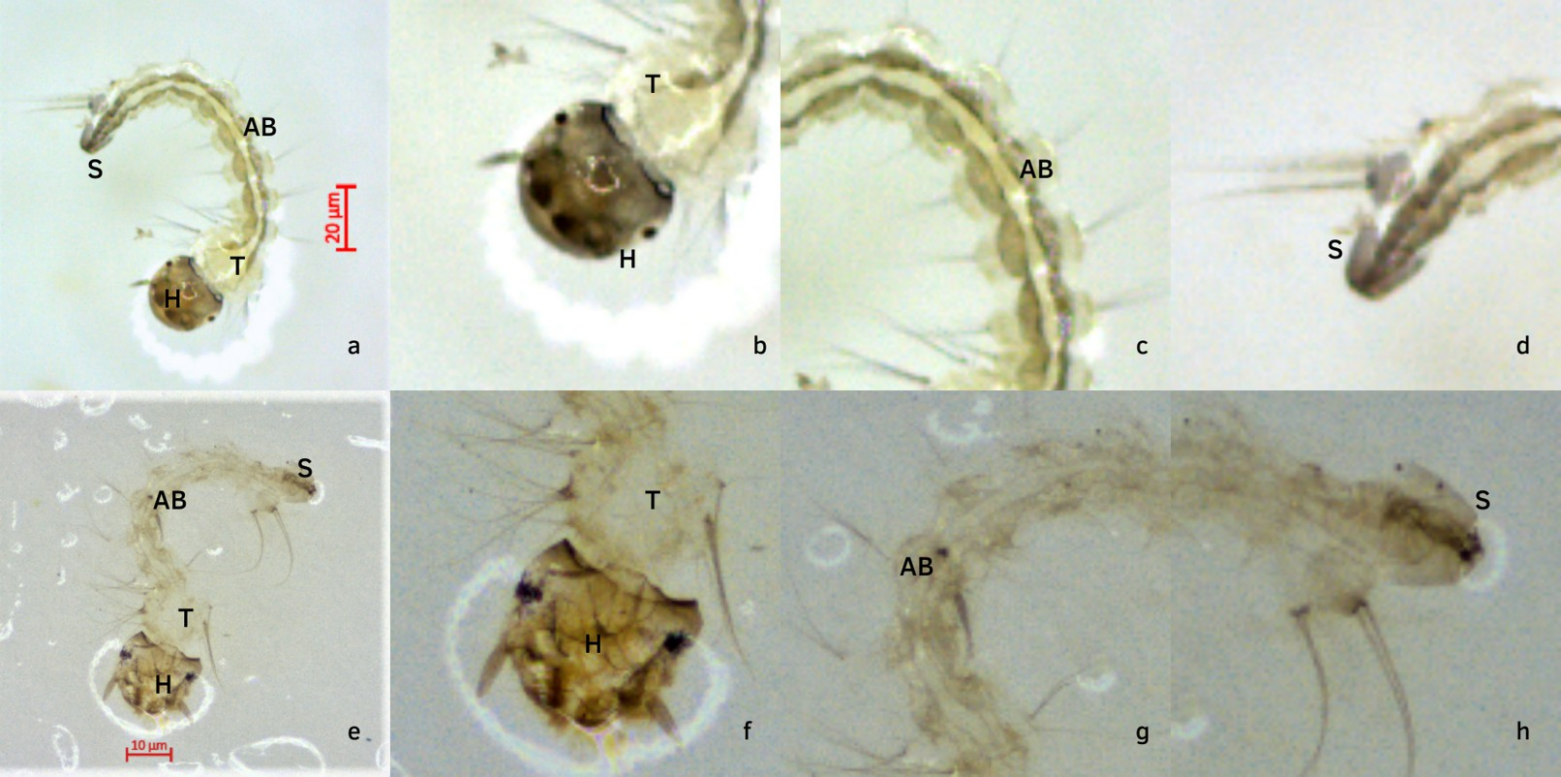
Alterations in morphological aspects of RHA-treated larvae. Concentration of extract (RHA): 0.3 mg/mL; Time of exposure: 24h; A-D: Control larva showing normal coloration of cuticle and others morphological aspects; e-h: RHA-treated larva showing discoloration of cuticle and morphological alterations; f: discoloration in the head (h) and thorax (t) of RHA-treated larva; g: abdomen (ab) discoloration of RHA-treated larva; h: few morphological changes in the siphon (s) of RHA-treated larva.

The larvicidal activity of RHA against *A*. *albopictus* larvae (n = 360 larvae) was determined. The LC_50_, LC_90_, and LC_99_ values (mg/mL) at 24 h (0.32, 0.70 and 1.32 mg/mL, Slope/SE = 3.846/0.067, χ ^2^ = 0.154 and R^2^ = 0.962) and 48 h (0.26, 0.47 and 0.78 mg/mL, Slope/SE = 4.855/0.059, χ ^2^ = 0.874 and R^2^ = 1.000) post-exposure were found to be similar (P = 0.1616) to those observed for *A*. *aegypti* larvae (Table 2). RHA-treated larvae showed discoloration a that started from the head and continued to the end of the abdomen, with few morphological changes in the siphon, in addition to a reduction in length (5.4 mm) compared to control larvae (6.7 mm) (Fig 1).

**Fig 2:**
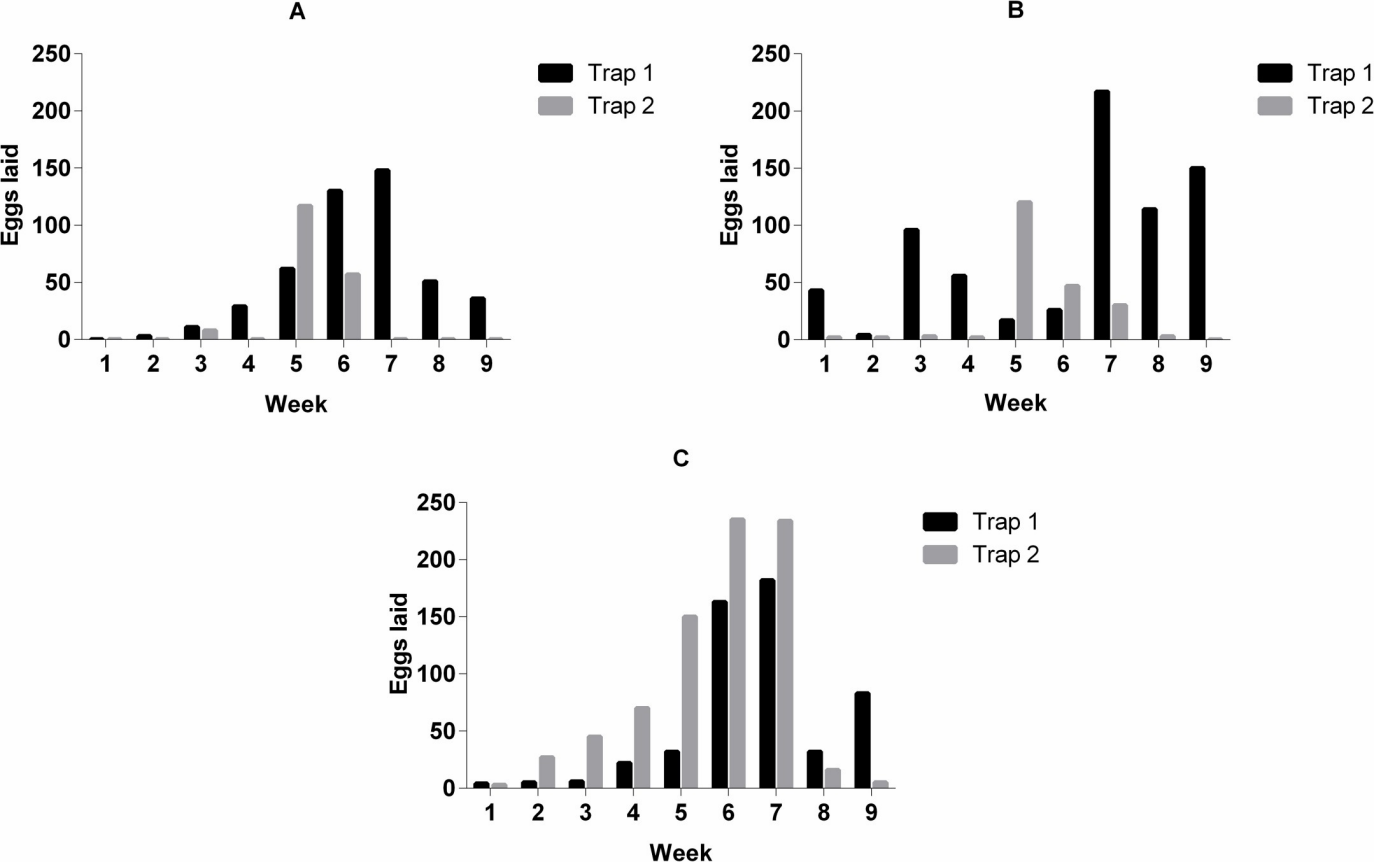
Effects of RHA on *Aedes* oviposition. Concentration of extract (RHA): 0.8 mg/mL. On the x-axis, there are the weeks of observation, and on the y-axis, the number of eggs laid by the *Aedes* females. A, B and C: Three points of high circulation of *A*. *aegypti*, at Universidade Estadual do Rio Grande do Norte, Mossoró-RN, Brazil. Trap 1 only contained water throughout the experiment while trap 2 had water replaced by RHA after the seventh week of observation.

The field tests suggest that RHA actively interferes with the oviposition profile of *A*. *aegypti* at a diagnostic concentration of 0.84 mg/mL, which is equivalent to the LC_90_ value observed 24 h post-exposure. According to the analysis of the results, RHA acts as a potential oviposition deterrent in urban areas with a high incidence of *A*. *aegypti* (Fig 2). During the first six weeks of observation, 3,908 eggs (n = 432 wood pallets) were counted in the ovitraps containing water. The eggs were distributed as follows: 880 eggs at point A (OPI = 75%; EDI = 48.89), 1,144 eggs at point B (OPI = 100%; EDI = 47.67), and 1,584 eggs at point C (OPI = 100%; EDI = 72.00). In the following three weeks, 2,026 eggs (n = 216 wood pallets) were counted in ovitraps containing water. The eggs were distributed as follows: 470 eggs at point A (OPI = 100%; EDI = 78.33), 962 eggs at point B (OPI = 100%; EDI = 160.33), and 594 eggs at point C (OPI = 100%; EDI = 99.00). However, as shown in Fig 2, in ovitraps containing RHA, a marked reduction was observed in the laying of eggs, with only 156 eggs (n = 216 wood pallets) observed over the three weeks (Point A: 0 eggs, OPI = 0% and EDI = 0; Point B: 66 eggs, OPI = 66.67% and EDI = 16.50; and Point C: 90 eggs, OPI = 100% and EDI = 15.00). During the entire study period (nine observation weeks), 6,090 eggs were counted and distributed in 864 wood pallets. As shown in Fig 2, the weekly count of laid eggs revealed the effectiveness of RHA in the first application, which reduced the number of eggs laid in ovitraps by approximately 90% compared to the water controls.

**Fig 3:**
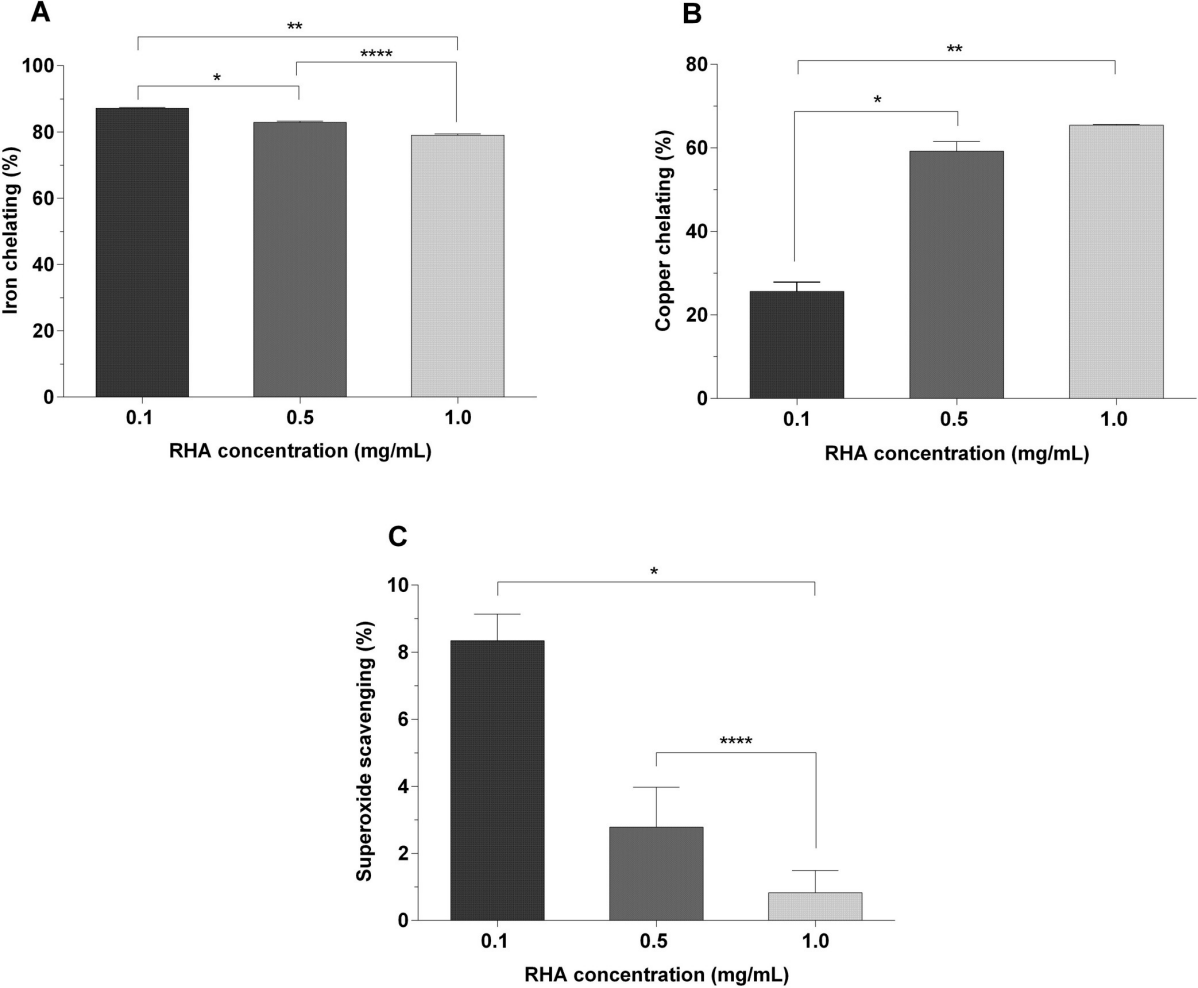
*In vitro* determination of the antioxidant activity of RHA. Concentration of extract (RHA): 0.1 to 1.0 mg/mL. On the x-axis, there are the RHA concentrations (in mg/mL), and on the y-axis, the percentage of antioxidant activity. Results expressed as mean ± standard error of the mean (n = 3). A: Iron chelating assay; B: Copper chelating assay; C: Superoxide scavenging assay. *: Statistical significance.

In addition to bioinsecticide activity, the antioxidant potential of RHA was investigated in several *in vitro* assays, apparently observed as a dose-response effect (Fig 3). The TAC of RHA was 286.86 mg EAA/g at a concentration of 0.1 mg/mL, indicating its applicability as an antioxidant compound. The iron-chelating capacity was directly proportional to the RHA concentrations employed, and a statistical significance in the effect was observed between all concentrations (P = 0.0001 to 0.03). At a concentration of 0.1 mg/mL, the iron-chelating capacity of RHA was 87.16%. The copper-chelating activity was directly proportional to the concentrations of RHA and varied between 25.64% and 65.45% for the lowest (0.1 mg/mL) and highest (1.0 mg/mL) concentrations investigated, respectively. However, no statistical significance (P = 0.9261) was found between 0.5 and 1.0 mg/mL in the copper chelating assay. At a concentration of 0.1 mg/mL, RHA demonstrated almost 10% antioxidant activity in the superoxide scavenging assays, with no statistical significance (P = 0.6732) relative to the activity observed at 0.5 mg/mL. However, at the maximum concentration investigated (1.0 mg/mL), the ability to scavenge superoxide radicals was lost (0.40% of activity). In the concentration range tested (0.1 to 1.0 mg/mL), RHA could not demonstrate antioxidant activity in the reducing power and hydroxyl scavenging assays.

**Fig 4:**
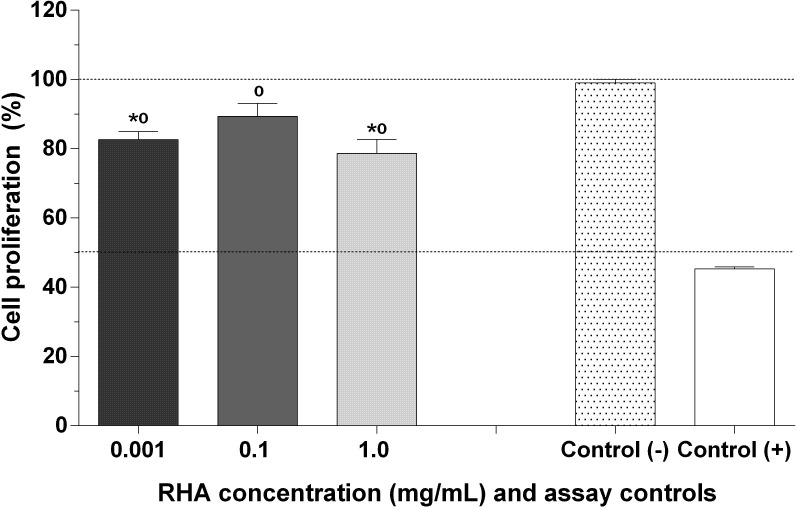
Antioxidant activity of RHA in cell culture. Concentration of extract (RHA): 0.001, 0.1 and 1.0 mg/mL. On the x-axis, there are the RHA concentrations (in mg/mL) and assay controls, and on the y-axis, the percentage of cell proliferation. Results expressed as mean ± standard error of the mean (n = 3). *: Statistical significance with the negative control;°: Statistical significance with the positive control. Control (+): 3T3 cells with reduced metabolic activity by up to 45% after exposure to H_2_O_2_ (4 mM); control (-): 3T3 cells without exposure to H_2_O_2_.

Despite the fact that *in vitro* assays indicate RHA to be an antioxidant agent in the chelation of metal ions, its effects in models that mimic RHA behavior on cell metabolism must be determined. As illustrated in Fig 4, RHA (0.001, 0.1 and 1.0 mg/mL, P = 0.8582) exhibited antioxidant activity in cell culture assays, promoting the viability of cells exposed to oxidative stress induced by H_2_O_2_ under experimental conditions. In particular, at a concentration of 0.1 mg/mL, RHA stimulated changes in the metabolic state of 3T3 cells, demonstrating cellular regeneration of 47% (92% cell viability), compared (P = 0.03) to the positive control (45% cell viability), following oxidative stress induced by H_2_O_2_.

In the range of concentrations tested (serial dilutions from 1024 to 32 μg/mL), RHA did not display antimicrobial activity against bacteria (*B*. *subtilis*, *E*. *coli*, *K*. *pneumoniae*, *P*. *aeruginosa*, *P*. *mirabilis*, *S*. *aureus*, *S*. *enterica* and *S*. *epidermidis*), yeast (*C*. *albicans*, *C*. *krusei*, *C*. *parapsilosis* and *C*. *tropicalis*) or filamentous fungi (*A*. *flavus*, *A*. *fumigatus*, *P*. *citrinum* and *R*. *oryzae*) investigated. However, RHA exhibited antileishmanial activity (IC_50_ = 3.53 mg/mL) by inhibiting the growth of promastigote forms of *L*. *amazonensis* by 23.71%, 16.67%, and 2.67% at concentrations of 1.0, 0.5, and 0.1 mg/mL (Slope/SE = 1.228/0.214, χ ^2^ = 0.932, R^2^ = 0.994, P = 0.001)_,_ respectively.

**Fig 5:**
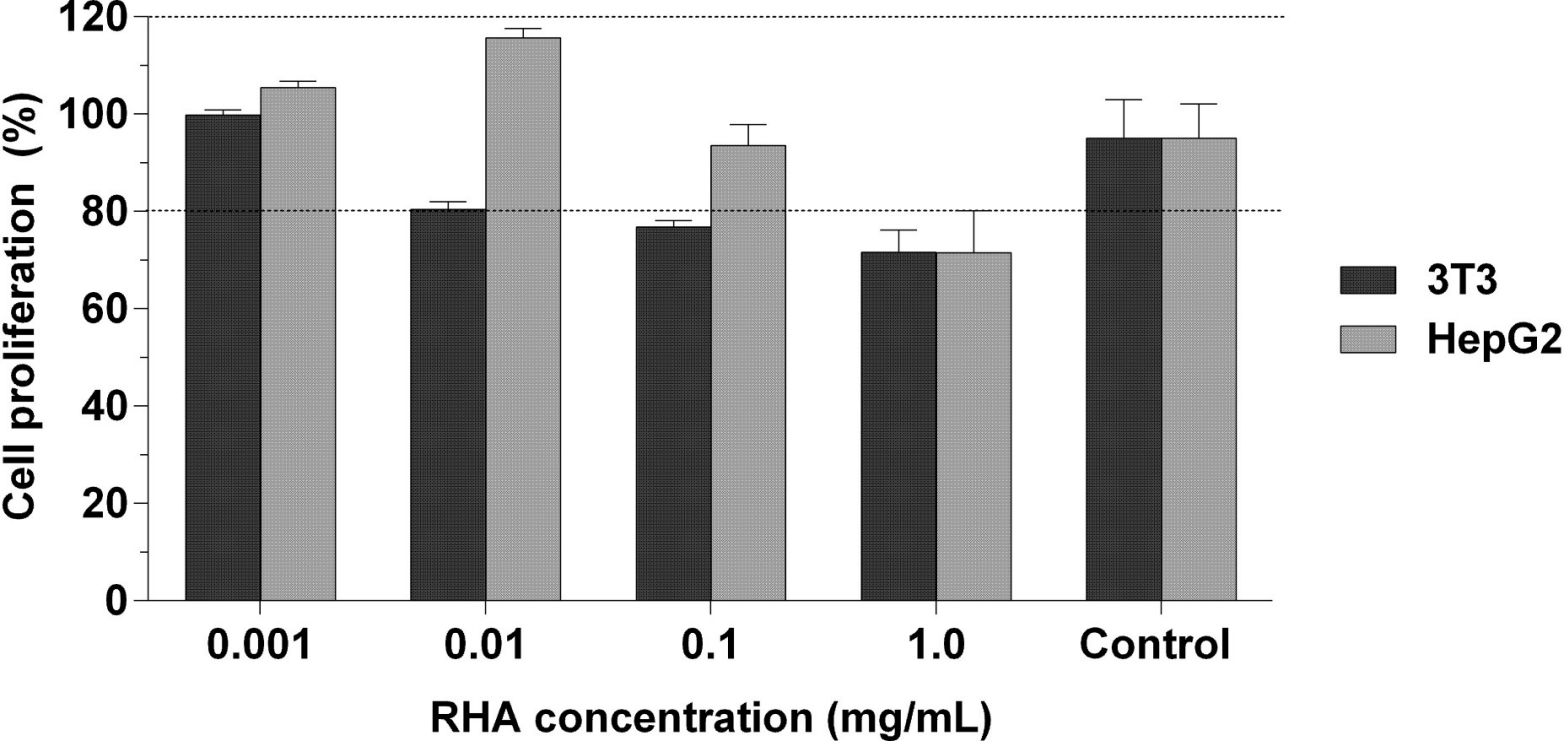
Cytotoxicity of RHA against 3T3 and HepG2 cells. Concentration of extract (RHA): 0.001 to 1.0 mg/mL. On the x-axis, there are the RHA concentrations (in mg/mL) and assay control, and on the y-axis, the percentage of cell proliferation. Results expressed as mean ± standard error of the mean (n = 3).

The results of toxicity analyses of RHA against the metabolic activity of HepG2 and 3T3 cells (Fig 5) and germination, growth, and biomass of *L*. *sativa* (Table 3) are indicative of its cytotoxic and environmental safety, respectively. Compared to the positive control (100% cell viability) of cytotoxic assays, no statistical significance (P = 0.2583) was found between all investigated RHA concentrations (0.001 to 1.0 mg/mL), indicating a tendency of low interference in the proliferation of 3T3 (CC_50_ = 5.07 mg/mL) and HepG2 (CC_50_ = 2.68 mg/mL) cells. This finding is interesting as RHA did not compromise the metabolic activity of these important cell lines in the range of concentrations with biological activity. *Lactuca sativa* germination (n = 16 germinated seeds) in RHA and distilled water (control) did not differ significantly to each other. Furthermore, up to the concentration of 0.3 mg/mL, RHA demonstrated the following parameters: first germination count (15 to 16 germinated seeds, P = 0.9052), radicle length (3.07 to 3.58 cm, P = 0.3790), relative seed germination (95.31 to 98.44%, P = 0.1817), seedling length (2.76 to 3.02 cm, P = 0.4489), relative radicle growth (94.57 to 111.29%, P = 0.1886), germination index (92.76 to 109.46%, P = 0.1817), and biomass (0.0112 to 0.0114g, P = 0.1897). According to the normalized residual indices of germinated seeds (-0.016 to -0.047, P = 0.6889) and root elongation (-0.054 to 0.113, P = 0.1885), RHA exhibited low toxicity to *L*. *sativa*.

**Table 3:** Phytotoxic effects of RHA against *Lactuca sativa*.

Parameters	Control	RHA (0.001 mg/mL)	RHA (0.01 mg/mL)	RHA (0.1 mg/mL)	RHA (0.3 mg/mL)
**FGC**	16 (16–16) ± 0.00	15 (15–16) ± 0.43	15 (15–16) ± 0.43	16 (16–16) ± 0.43	16 (16–16) ± 0.43
**RL (cm)**	3.33 (2.675–4.10) ± 0.58	3.07 (3.07–3.22) ± 0.25	3.31 (3.13–3.46) ± 0.12	3.15 (2.86–3.43) ± 0.20	3.58 (3.35–3.91) ± 0.21
**RSG (%)**	na	98.44 (93.75–100) ± 2.71	95.31 (93.75–100) ± 2.71	98.44 (93.75–100) ± 2.71	98.44 (93.75–100) ± 2.71
**SL (cm)**	2.93 (2.66–3.23) ± 0.22	2.76 (2.58–3.00) ± 0.16	3.02 (2.84–3.13) ± 0.11	2.89 (2.68–3.22) ± 0.20	2.70 (2.24–3.26) ± 0.36
**RRG (%)**	na	94.57 (78.51–115.93) ± 14.65	102.01 (84.46–123.63) ± 15.51	97.19 (76.22–119.36) ± 15.43	111.29 (84.90–146.28) ± 23.23
**GI (%)**	100 ± 0.00	92.76 (78.51–108.68) ± 12.13	96.95 (84.46–115.90) ± 13.16	95.63 (76.22–119.36) ± 15.41	109.46 (84.90–146.28) ± 22.99
**BIO (g)**	0.0106 (0.0101–0.0101) ± 0.00	0.0112 (0.0106–0.0118) ± 0.00	0.0113 (0.0108–0.0118) ± 0.00	0.0113 (0.0106–0.0118) ± 0.00	0.0114 (0.0106–0.0120) ± 0.00
**NRPGSI**	na	-0.016 (-0.063–0.000) ± 0.03	-0.047 (-0.063–0.000) ± 0.03	-0.016 (-0.063–0.000) ± 0.03	-0.016 (-0.063–0.000) ± 0.03
**NRERI**	na	-0.054 (-0.215–0.159) ± 0.15	0.020 (-0.155–0.236) ± 0.16	-0.028 (-0.238–0.194) ± 0.15	0.113 (-0.151–0.463) ± 0.23

Concentration of extract (RHA): 0.001 to 0.3 mg/mL Results expressed as mean (lower–upper) ± standard error of the mean (n = 4). Control: Seeds of *Lactuca sativa* germinated in distilled water. Na: Not Applied; FGC: First Germination Count; RL: Radicle Length; RSG: Relative Seed Germination; SL: Seedling Length; RRG: Relative Radicle Growth; GI: Germination Index; BIO: Biomass; NRPGSI: Normalized Residual Percentage of Germinated Seeds Index; NRERI: Normalized Residual Elongation Root Index.

**Fig 6:**
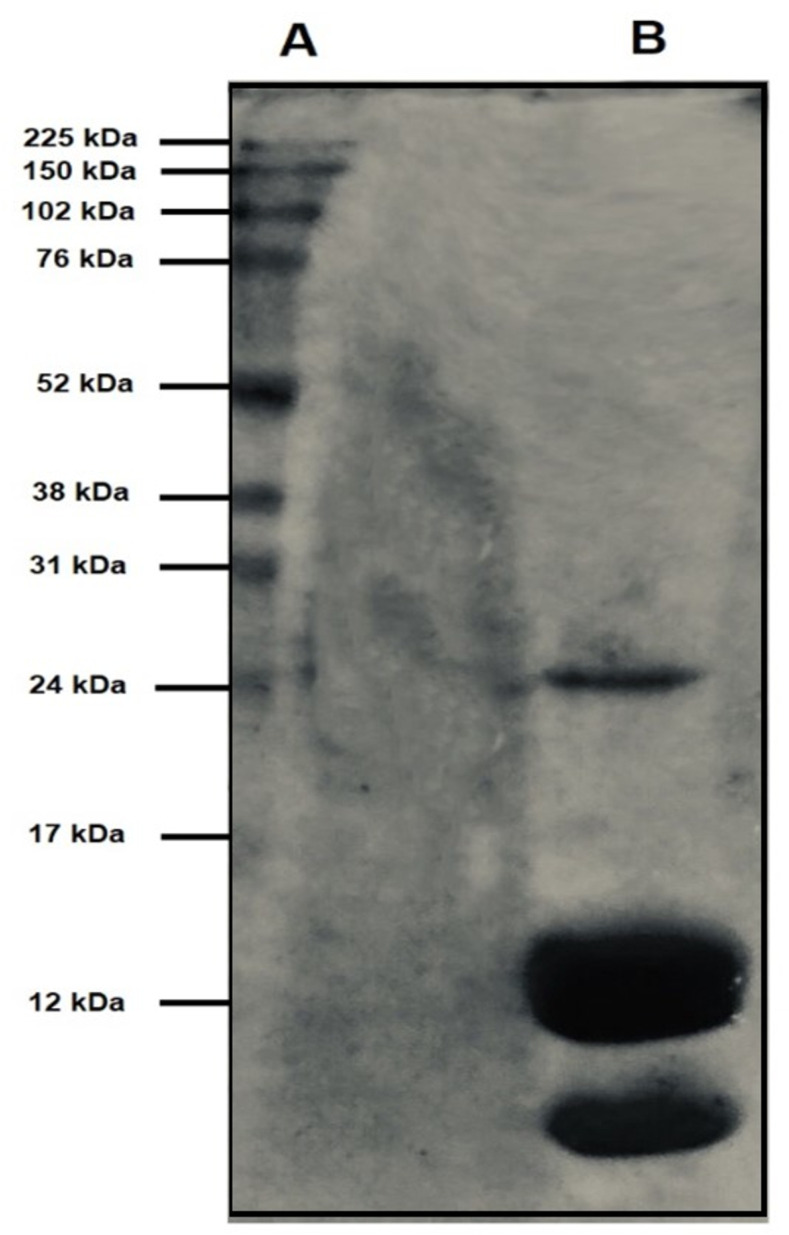
Electrophoresis (SDS-PAGE, 12.5%) of RHA. A: Molecular mass markers (225–12 kDa); B: Hydroethanolic extract from roots, RHA (30 μg).

The extraction conditions enabled the concentration of carbohydrates (38% ± 0.02), proteins (3.3% ± 0.02), and phenolic (0.2% ± 0.01) compounds in RHA. Although the levels of proteins extracted from RHA were lower than those of carbohydrates, a lack of studies on the protein diversity of *T*. *toxicaria* extracts has led to the examination of the protein profile of RHA. Electrophoresis of RHA revealed proteins with molecular masses between 10 and 24 kDa, with a peculiar concentration at 12 kDa (Fig 6). Among the possible classes of proteins derived from RHA, the analyses confirmed the presence of lectins; however, the expression was low (32 HU.100μL) and was observed under specific conditions (blood type A papain-treated). Additionally, four classes of protease inhibitors were detected, with percentage inhibition of enzymatic activity varying among the enzymes investigated (i.e., percentual inhibitions of bromelain = 89.08% ± 0.01; papain = 72.77% ± 0.00; chymotrypsin = 56.47% ± 0.01; and trypsin = 10.73% ± 0.01).

## Discussion

Among the larvicidal extracts obtained from *T*. *toxicaria*, the hydroethanolic extract from roots (RHA) was the most active (Tables 1 and 2), consistent with results presented by Bakar et al. [57] regarding the influence of extraction conditions and plant organs on extract bioactivity. Although most available studies employing *Tephrosia* formulations use their aerial parts as matrices suitable for obtaining insecticidal formulations [10, 58, 59], roots are also a promising source of insecticidal biomolecules [60, 61].

Analysis of the results suggests that the larvicidal compounds from RHA could compromise larval nutrition by inducing oxidative damage in the midgut of *Aedes* larvae after migration through the peritrophic membrane, resulting in larval death, similar to reported by Procópio et al. [62]. These authors [62] have demonstrated that exposure of *Aedes* larvae to *Schinus terebinthifolius* extract results in the disorganization of the larval midgut epithelium, which might be attributed to the accumulation of the extract in the larval midgut. Reports indicate that this phenomenon can be attributed to the high permeability of the larval peritrophic membrane [63]. The discoloration of RHA-treated larvae (Fig 1) may be a consequence of the interaction of insecticidal compounds from extract with larvae cuticle chitin, as suggested by De Araújo et al. [64]. Subahar et al. [65] attribute the reduction in the length of RHA-treated larvae to the larval exoskeleton damage, compromising the nutrient absorption and toxin excretion. Before death, RHA-treated larvae displayed lethargic behavior, i.e., reduced mobility and responses to the physical and mechanical stimuli. Inducing such behavior is one of the possible modes of action of botanical larvicides [66]. Thus, the suggested mode of action of RHA comprises induction of toxicity and oxidative stress in the larval midgut, and consequent morphological alterations, reduction of mobility and stimuli perception, and larval death.

Initial chemical characterization of RHA revealed a variable amount of carbohydrates, soluble proteins, and phenolic compounds. Most available studies suggest that *T*. *toxicaria* is enriched in carbohydrates and phenolic compounds, especially rotenoids [10, 11]. The low concentration of extracted protein in RHA (3.3%) may be associated with the extraction conditions, as the protein content is influenced by the type of organ plant and extraction method used. In particular, alcoholic extraction typically promotes low protein yield [67, 68], suggesting incomplete solubilization of proteins from RHA. The roots of terrestrial plants represent a complex system for transporting water and nutrients and are involved in plant anchoring, hormone synthesis, and storage [69]. However, these roots secrete proteolytic enzymes that digest proteins on the root surface, making it difficult to extract proteins from the plant organ [70]. High vacuolization of the roots and the presence of secondary metabolites also negatively affect the concentration and extraction of proteins [71]. Together, these factors may have compromised a better concentration of soluble proteins in RHA.

Although these limitations compromise the concentration of proteins in plant root extracts, the method used for obtaining RHA was effective in extracting proteins with few technological resources, processes, and reagents, while enabling easy reproducibility. To the best of our knowledge, studies on the characterization of the proteins in *T*. *toxicaria* extracts have not been conducted. Hence, to begin the characterization of proteins extracted from RHA, protease inhibitors and lectins were investigated. Cysteine protease inhibitors were expressed in higher proportions than serine protease inhibitors, inhibiting the activity of bromelain, papain, chymotrypsin, and trypsin enzymes by 89.08%, 72.77%, 56.47%, and 10.73%, respectively. RHA extraction could also fractionate lectins even with low hemagglutination titers (32 HU.100 μL).

Proteases are enzymes that play crucial roles in various biological and physiological processes [72]. The proteolytic activity of proteases is tightly regulated by protease inhibitors [72]. In legumes, cysteine protease inhibitors (an abundant inhibitor class in RHA) typically demonstrate molecular masses between 11 and 23 kDa [73]. Lectins are glycoproteins that can reversibly bind specifically to the surface carbohydrates of a target cell, promoting its precipitation or agglutination [74]. Legume lectins can form homodimers or homotetramers in solution with molecular masses around 30 kDa [74]. The electrophoretic profile of RHA (Fig 6) revealed the presence of proteins with molecular masses between 10 and 24 kDa, thereby reinforcing the hypothesis that cysteine protease inhibitors with molecular masses of approximately 12 kDa and lectins with molecular masses of approximately 24 kDa are components of the bioactive molecules of the extract.

Numerous studies have suggested that botanical formulations containing protease inhibitors and lectins may exert a detrimental effect on the development of *Aedes* larvae [75–78]. These studies proposed that protease inhibitors and lectins act by inhibiting biomolecules that play an essential role in larval nutrition or compromising the structure of midgut, and by extension midgut physiology, which may justify the morphophysiological changes observed in response to RHA treatment. However, we cannot rule out the hypothesis that RHA activity can be attributed to the synergistic effect of proteins and other compounds that are present in the extract. Vasconcelos and coworkers [10] fractionated the ethanol extract of *T*. *toxicaria* roots, and identified three classes of rotenoids with larvicidal activity, namely, deguelin, 12a-hydroxy-α-toxicarol, and tephrosin. Vasconcelos et al. [10] reported that the structure-activity relationship of the rotenoids could not be confirmed, suggesting the influence of the lipophilicity of the rotenoids in the manifestation of larvicidal activity.

The bioactivity of RHA against different species of *Aedes* larvae can be leveraged for using RHA in different environments, especially as *A*. *aegypti* is well adapted to urban areas, especially human homes [79], while *A*. *albopictus* is commonly found in urban and rural environments [80]. Unfortunately, despite evidence regarding the bioinsecticide efficacy of plant extracts in laboratory settings, only a few studies have been performed to validate their efficacy in the field. In this study, the performance of RHA as a bioinsecticide was investigated by placing the extract in ovitraps distributed at three points in the Universidade do Estado do Rio Grande do Norte (UERN), Mossoró-RN, Brazil. The entomological control team at UERN reported that the campus had a high incidence of *A*. *aegypti* (data regarding the occurrence of other insect species were not provided). More than 73,000 *A*. *aegypti* eggs were collected and identified between August 2017 and July 2018 at 16 monitoring points on the campus.

Based on OPI (75%– 100%) and EDI (47.67–160.33) values, was confirmed the presence and reproductive activity of adult *A*. *aegypti* females in all surveyed areas at observational weeks (Fig 2). Costa et al. [81] reported that the reproductive performance of *A*. *aegypti* is influenced by climatic factors, with high fertility rates under conditions of high temperature and humidity, which are similar to the averages found in Mossoró during the investigation period (temperature: 27.8°C; humidity: 77.1%). The association between climate variables and other covariates and the spatiotemporal distribution of the *A*. *aegypti* population is the subject of ongoing research to determine the insecticidal effects of RHA at concentrations equivalent to the LC_50_ value at 24 h (0.3 mg/mL).

The positivity of ovitraps in UERN suggests that the investigation sites in this study were constantly under alert for the incidence of arboviruses. Epidemiological bulletins from the State Government of Rio Grande do Norte, Brazil, reported 451 cases of dengue and 202 cases of chikungunya in Mossoró city between 12/29/2019 and 03/14/2020 (Epidemiological Weeks 01 to 14) [82]. Although vector control activities are employed by municipal authorities, a constant occurrence of females in the investigated locations can be assumed; such occurrence promotes the onset of arbovirus outbreaks. These data suggest the insufficiency of the applied interventions and the need to implement adjuvant management strategies. Thus, according to WHO [29] recommendations, a diagnostic concentration corresponding to the LC_90_ (0.8 mg/mL) value of RHA obtained under laboratory conditions following 24 h of exposure was defined to assess the insecticidal effect of the extract in this area.

Analyses of RHA (0.8 mg/mL) performance suggest its use as a potential oviposition deterrent as a smaller proportion of eggs was laid in ovitraps containing RHA than that in the water controls (Fig 2). Dethier et al. [83] define deterrent (also called antifeeding, suppressant, anorexigenic and anti-appetant) as a chemical that inhibits feeding or oviposition when present in a place where insects feed or oviposit. Oviposition is one of the most important events in the mosquito life cycle [84], and our results (Fig 2) demonstrate that RHA interferes with this process. The reproductive cost associated with RHA may be attributed to interference in the reception of olfactory or taste stimulants, which are essential for directing pregnant females to potential oviposition sites. This inference is in line with that observed in studies examining interference in olfactory or taste responses in response to botanical insecticides [85, 86]. The results of these studies offer possibilities for the incorporation of botanical insecticides in current vector management systems instead of chemical insecticides.

Female insects are expected to fly in opposite directions from traps containing RHA (hypothesis based on the absence of egg laying in ovitraps containing the extract). However, if females lay eggs in traps containing RHA, the larvae will die after hatching as RHA exhibits larvicidal activity. These results demonstrate the versatility of plant-derived extracts, such as RHA, and provide more options for insect control programs. Field studies in Tanzania and Malawi [87] demonstrate the efficacy of *Tephrosia* extracts in controlling agronomic pest populations. In India, after treating sewer systems with plant extracts, the larval density of *Culex quinquefasciatus* was significantly reduced [88]. Thus, the detrimental effects of RHA on insect oviposition and its safety profiles (both cytotoxic, Fig 5, and environmental, Table 3) are of particular interest for developing insect management formulations. Promising results for the control of field pests and arbovirus vectors using RHA-based nanostructured formulations have already been obtained by our group. Our results have also resulted in two patent applications [89, 90] at the National Institute of Industrial Property (Brazil).

Although ecologically friendly options are emerging, the use of chemical insecticides remains the most common strategy for the management of arboviruses. However, chemical insecticides influence important developmental events in the lifecycle of non-target organisms, and their residues can persist in edible vegetables, thereby seriously affecting food and environmental safety [91]. The identification of natural insecticides that exhibit low toxicity against non-target organisms, such as other plants, is a biotechnological objective to overcome this weakness. The phytotoxic effects of 0.001 to 0.3 mg/mL RHA (Table 3) do not compromise the initial development and biomass of the non-target plants (*L*. *sativa*), and the investigated toxicity indices (NRPGSI and NRERI) suggest that RHA can be used as an ecologically safe formulation.

The antioxidant potential of RHA has been confirmed in different trials. The total antioxidant capacity (TAC) of RHA was 286.86 mg AAE/g, a value higher than that of *Myrciaria tenella* (0.013–0.020 mg AAE/g) and atemoya (51–94 mg AAE/g) extracts [92], when tested at the same concentration (0.1 mg/mL). TAC refers to the content of oxidants (moles) eliminated by the antioxidant compound and is a reference for the quantification of total antioxidant content [93]. Thus, RHA is an excellent source of antioxidant molecules. Other *in vitro* assays (Fig 3) confirmed the antioxidant properties of RHA by demonstrating iron and copper chelating and superoxide scavenging capacities, as a dose-response reflex to the RHA concentrations employed. Similar to the effects of bioinsecticides, many studies have described the antioxidant benefits of rotenoids [94], lectins [95] and protease inhibitors [96] derived from plant formulations. These studies demonstrate the therapeutic and biotechnological relevance of plant extracts, reinforcing the hypothesis that the biological activities of RHA can be promoted by a complex mixture of non-protein compounds and proteins classically associated with plant defense mechanisms against pests, pathogens and abiotic factors.

At the lowest concentration of RHA (0.1 mg/mL), the iron-chelating capacity was 87.16% (Fig 3). Although iron is crucial for several enzymatic pathways that involve redox reactions [97], under conditions of imbalanced homeostasis, it becomes pathogenic by producing reactive species, potentiating the development of Alzheimer’s and Parkinson’s diseases [98], and inducing hepatic [99] and neurotoxic [100] complications. To date, no cure is available for diseases involving iron overload, and the available therapeutic options include controlling iron levels in the body by administering chelating compounds. The extracts of *Gundelia tournefortii* [101] and *Epilobium hirsutum* [102] have been reported to improve the serum iron profile and function in experimental rats; this reinforces the use of RHA as a new prospective source of natural antioxidants that can expand the therapeutic repertoire for complications related to iron overload.

The copper-chelating activity of RHA was between 25.64% and 65.45% for the lowest (0.1 mg/mL) and highest (1.0 mg/mL) concentrations investigated, respectively (Fig 3). Similar to iron, copper is an essential micronutrient for the proper functioning of the human body [103]; however, when it is not properly metabolized, copper bioaccumulates in cells and promotes numerous neurodegenerative disorders and syndromes [104]. RHA-based formulations may be good adjuvants to current therapies to control the copper levels, as Nguyen et al. [105] suggested that plant extracts could be used to treat copper-induced oxidative damage. RHA may also serve as an alternative for use in agriculture, as previous studies [106] have revealed promising results for the use of plant extracts in recovery soils contaminated with potentially toxic metals. The low activity in superoxide scavenging assays (Fig 3) and the absence of antioxidant activity in the hydroxyl scavenging and reducing power assays suggest that RHA exhibits selective antioxidant activity.

An extremely important aspect in evaluating the safety and toxicity of plant extracts is their impact on cell metabolism. In this study, we evaluated the cytotoxic effects of RHA in two cell lines (murine fibroblast and hepatocellular carcinoma cells, 3T3 and HepG2, respectively) using the MTT assay, a simple and rapid colorimetric assay [107]. The principle of the MTT assay involves the reduction of tetrazolium salt to formazan crystals, and the concentration of dissolved crystals is directly correlated with the number of metabolically active cells [108]. The findings of this assay demonstrate the cytotoxic safety of RHA (Fig 5) as the extract was not found to compromise the metabolic activity of cells compared to the control cells (P = 0.2583). HepG2 cell lines are often used in *in vitro* studies of drug metabolism owing to their compatibility with primary human hepatocytes [109]. The absence of cytotoxicity for this line cell suggests the possible hepatic metabolism and consequent cytotoxic safety of RHA in therapeutic and biotechnological applications.

RHA did not compromise the metabolism of 3T3 cells (Fig 5), and this result was critical for evaluating the effects of RHA on H_2_O_2_-induced oxidative stress in cell culture. In this model, cell proliferation was interrupted, and the accumulation of molecular factors associated with senescence was promoted [110]. The antioxidant activity of RHA may be associated with its antioxidant components and the recruitment of essential cell molecules to the reestablishment of mitochondrial potential, resulting in the restoration of the metabolic activity of 3T3 cells, an important fibroblast cell model; this is similar to the mechanism proposed by Pieńkowska et al. [111]. Because the regulation of the expression of genes that encode proteins involved in the inactivation of reactive species has not been followed, the exact mechanism underlying these events needs to be determined in further studies. The redox homeostasis of fibroblasts is essential for healing processes, as changes in their status impair the activity of enzymes involved in this process [112] and trigger even more drastic effects during the healing of diabetic wounds. The preliminary results of this study are essential indicators for understanding the potential effects of RHA on the oxidative status of important cells, such as fibroblasts, and promising formulations with therapeutic potential in regenerative medicine.

The implementation of natural plant formulations in regenerative medicine is already a viable possibility [113, 114]. Since 1981, approximately 40% of the drugs approved by the FDA have been obtained from natural sources, including plants [2]. Other public policies, such as the Brazilian Policy on Medicinal Plants and Phytotherapeutics, also recognize the importance of developing actions aimed at safe access and rational use of medicinal and phytotherapic plants [115]. Based on the results of this study, *in vivo* tests can be designed to assess the antioxidant potential of RHA on cellular metabolic reprogramming in complications induced or associated with oxidative stress. RHA-based formulations can be obtained and optimized in systems that do not exhibit malabsorption or rapid/inadequate metabolism of bioactive compounds.

In serial dilution experiments (1024–32 μg/mL), RHA did not show antimicrobial activity against investigated bacteria, yeast, or filamentous fungi. Despite studies confirming the antimicrobial activity of alcoholic extracts from other *Tephrosia* species, it has an MIC > 2.5 mg/mL [116, 117], reaching MIC = 200 mg/mL [118]. These results suggest that the antimicrobial formulations of *Tephrosia* must be administered in high concentrations for the manifestation of their activity. Antipromastigote activity, with inhibition of *L*. *amazonensis* growth by 24%, was observed at an RHA concentration of 1.0 mg/mL. The antileishmanial activity of RHA was considered weak because the IC_50_ (3.53 mg/mL) was greater than 100 μg/mL, according to the Cos et al. [119] criteria. However, Gertsch [120] argues that ethnopharmacological research should provide new insights into plant pharmacology to develop adjuvant therapeutic options for numerous infectious complications. The bioinsecticide and antioxidant activities of RHA were potent at low and safe concentrations, confirming its effectiveness and potential in the development of products that can support for the integrated management of vectors and regenerative medicine.

Prospecting and exploring the potential of vegetation occurring in the northeast of Brazil via sustainable approaches are effective strategies for better interaction and valuation of northeast ecosystems, which can represent an improvement in the quality of life and income of local communities [121, 122]. Studies on mutagenic, genotoxic, and indirect toxic effects on terrestrial and aquatic ecosystems are underway to expand understanding of the RHA safety as an adjuvant for insect and disease management policies.

## Conclusions

Among the eight extracts of *T*. *toxicaria*, the hydroethanolic extract from roots (RHA) was identified as the multivalent insecticidal extract, with larvicidal and oviposition deterrent activities, in addition to antioxidant and antileishmanial activities. Initial chemical characterization revealed that RHA extraction was efficient at concentrating carbohydrates, phenolic compounds, and proteins, especially protease inhibitors and lectins. The promising results associated with biological activities (insecticidal and antioxidant) and low toxic effects (cytotoxic and phytotoxic) indicate that RHA is a suitable candidate for the development of adjuvant formulations for insect and disease management protocols. Greater incentives and investment in research associated with the flexibility of regulatory policies are essential for a more agile implementation of safe and effective botanical extracts in insect and disease management policies. Importantly, the involvement of the pharmaceutical industry is critical to the discovery of new treatments for tropical and chronic diseases.
